# Palliative Treatment for the Management of Advanced Pelvic Hydatid Bone Disease

**DOI:** 10.4269/ajtmh.23-0267

**Published:** 2023-07-31

**Authors:** Haopeng Luan, Kai Liu, Qi Tian, Yuanxin Chen, Cong Peng, Xiaoyue Sun, Xinghua Song

**Affiliations:** ^1^ Department of Spine Surgery, The Six Affiliated Hospital of Xinjiang Medical University, Urumqi, Xinjiang, China;; ^2^ Department of Trauma and Microreconstructive Surgery, The First Affiliated Hospital of Xinjiang Medical University, Urumqi, Xinjiang, China;; ^3^ Department of Bone Tumor Surgery, The First Affiliated Hospital of Xinjiang Medical University, Urumqi, Xinjiang, China;; ^4^ Uygur Medical College, Xinjiang Medical University, Urumqi, Xinjiang, China;; ^5^ Department of Rehabilitation Medicine, Shenzhen People’s Hospital, Shenzhen, Guangdong, China

## Abstract

Hydatid bone disease is a zoonotic parasitic infection that is caused primarily by the tapeworm *Echinococcus granulosus*, and it continues to be a major public health concern in pastoral regions. The reconstruction of limb function after limb salvage surgery remains a challenge for clinicians. The purpose of this study was to determine the clinical efficacy of palliative treatment of the management of advanced pelvic hydatid bone disease. From March 2005 to December 2018, medical records and images of patients with advanced pelvic hydatid bone disease treated with surgery combined with antiparasitic chemotherapy were evaluated retrospectively. The Enneking classification was applied to determine the location of the lesion, and the Musculoskeletal Tumor Society score system was used for outcome evaluation. Fifteen patients who met the criteria were included in this study, with a mean follow-up of 4.40 ± 1.76 years. All patients received treatment with surgery combined with antiparasitic chemotherapy. The mean number of surgical interventions per patient for pelvic cystic echinococcosis was 5.3 (range, 2–9 interventions per patient). Recurrence of pelvic hydatid bone disease occurred in 5 patients and was managed successfully through repeated debridement procedures. Palliative treatment with limb salvage surgery was an effective and practical approach to the management of advanced pelvic hydatid bone disease. Standard antiparasitic chemotherapy, which included albendazole at a dose of 10 mg/kg/day administered in two daily doses for 3 to 6 months, was also considered an essential part of the overall treatment strategy.

## INTRODUCTION

Hydatid bone disease is a zoonotic parasitic infection caused primarily by the tapeworm *Echinococcus granulosus*, and it presents a significant public health concern in pastoral regions of developing countries, including North Africa, southern Russia, central Asia, and western China.^1^^–^^3^ Cystic echinococcosis (CE) commonly invades the liver and lungs (∼90% of cases) and, as a result, the incidence of bone CE (0.5–4% of total CE) is expected to increase proportionally.^4^^–^^6^ The most common site of bone lesions invaded by CE is the vertebral column (40–50%), followed by large bones (25–30%), the pelvis (15–20%), and, less frequently, the cranium, sternum, scapula, and phalanges.^7^^–^^9^ Notably, the growth and development of echinococcosis is slow and involves major invasion of bone trabeculae, resulting in delayed manifestation of typical symptoms, which take many years to appear. The insidious and distinctive nature of CE makes it difficult to diagnose and treat, often resulting in severe dysfunction or even death.^1^^,^^10^

The biological behavior of pelvic echinococcosis is more complex than that of spinal hydatid disease, often resulting in misdiagnosis as tumors or tuberculosis, and thus more severe bone destruction. Treatment of pelvic hydatid bone disease currently comprises surgical resection and reconstruction combined with antiparasitic chemotherapy.^4^^,^^6^ The principle of surgery is to ensure the complete resection of the cyst below the surgical border, restoring pelvic stability with simple implants. Despite careful resection to avoid rupture of the cyst during surgery, residual cysts and recurrence remain common.^9^^,^^11^ The reconstruction of limb function after limb salvage surgery also remains a challenge for clinicians. Recently, artificial hemipelvic prostheses have been suggested as a practical option for limb function when lesions involve more than two regions of the pelvis (Enneking classification).^6^ The purpose of this study was to evaluate the outcomes and complications of palliative treatment of the management of advanced pelvic hydatid bone disease.

## MATERIALS AND METHODS

### Study design.

From March 2005 to December 2018, a retrospective evaluation was conducted on medical records and imaging of patients with pelvic hydatid bone disease who were treated by surgery combined with antiparasitic chemotherapy. The inclusion criteria for the study were as follows: 1) confirmation of pelvic hydatid bone disease by computed tomography (CT) showing round or ovoid space-occupying lesions with double-layer arcuate calcification, and magnetic resonance imaging (MRI) showing multiple cystic fluid-filled lesions separated with thin walls and irregular branching, resembling a bunch of grapes at multiple levels; 2) positive results from serological tests for echinococcosis, such as ELISA and dot immunosphere filtration assay (DIGFA); 3) histological evidence supporting pelvic echinococcosis; 4) presence of pain or restricted movement in the hip or gluteal region; and 5) treatment with surgery combined with antiparasitic chemotherapy. Exclusion criteria included incomplete medical records, poor compliance, or a follow-up of less than 20 months.

A hemipelvic prosthesis was then designed on a computer, with its articular surface anatomically apposed to the auricular surface of the sacrum on the lesion side of the pelvis. Antiparasitic chemotherapy using albendazole at a dose of 10 mg/kg/day (given in two daily doses) for 3 to 6 months was managed preoperatively to reduce cyst size and avoid spreading.^3^ Indications for surgical intervention included persistent back pain, nerve or organ compression symptoms, and an imbalanced pelvis caused by osseous destruction that could not be relieved after antiparasitic chemotherapy. The diagnosis was established based on the results of preoperative portable ultrasound and intraoperative histological analysis of the lesion.

### Surgical technique.

Resection of pelvic regions I and II or I, II, and III was conducted according to the extent of echinococcosis involvement. The basic technique involved the protection of adjacent normal tissue with gauze soaked in 20% sodium chloride solution, followed by cleaning the interior wall of the residual cavity using a high-speed burr. Osteotomy was carried out through the sacroiliac articular surface when resection was managed in pelvic region I. The sacral articular surface of the preoperatively printed hemipelvic prosthesis was precisely shaped to fit the sacral auricular surface. The wing of the prosthesis was supported by the lower sacrum, and screws were carefully placed in the predetermined screw holes to firmly secure the prosthesis. To enhance its stability, bone cement was applied from the sacroiliac joint to the superior acetabulum after the pelvic prosthesis was fixed in position. Intraoperative care was taken to avoid lumbosacral nerve compression.

### Postoperative management.

All patients received treatment with albendazole at a dose of 10 mg/kg/day, administered in two daily doses for a period of 3 to 6 months.^3^ In cases of recurrence, the dose was increased to 10 to 12 mg/kg/day for 1-month courses, followed by a rest period of 15 days after each month. Liver and kidney function were monitored every 6 weeks, whereas radiography and blood tests were performed at 1, 3, 6, 9, 12, 18, and 24 months after surgery. Enneking classification^12^ was applied to determine the location of the lesion, and the Musculoskeletal Tumor Society (MSTS) score system was used for outcome evaluation.

### Statistical analysis.

Data were input in an Excel 2016 spreadsheet (Microsoft Corp., Redmond, WA) and were analyzed using SPSS 20.0 software (SPSS Inc., Chicago, IL). Continuous variables were expressed as mean ± SD.

## RESULTS

Fifteen patients were included in this study, with a mean follow-up of 4.40 ± 1.76 years. The affected regions of the pelvis in all patients are presented in Table 1, and were confirmed by the results of CT and MRI. Typical poisoning symptoms of the parasitic disease (back pain and progressive weakness) were observed in all patients. Preoperative imaging (CT and MRI) and positive results from parasitic immunodiagnostic tests confirmed that 14 patients had CE and one patient had alveolar echinococcosis, which was also supported by intraoperative histological findings. Extraosseous involvement was observed in eight patients (53.3%), including five with hepatic infection and three with pulmonary infection.

**Table 1:** Patient data

No.	Gender	Age, years	Enneking classification	Extrapelvic infection	Treatment	Follow-up, years	MSTS score at final follow-up	Outcome
1	M	45	I, II, and right hip joint	Hepatic infection	RD + HR + AHP + FPR + AC	5	29	FOD
2	M	30	I, II, III, and left hip joint	Hepatic infection	RD + HR + AHP + AHP + AC	7	20	FOD
3	M	46	I, II, IV, and right hip joint	None	RD + HR + AHP + FPR + AC	8	21	FOD
4	F	39	I, II, III, IV, and left hip joint	Hepatic infection	RD + HR + AHP + FPR + AC	4	23	HP
5	F	54	I, IV, and left hip joint	None	RD + HR + AHP + FPR + AC	4	18	FOD
6	F	54	I, II, IV, and right hip joint	None	RD + HR + AHP + FPR + AC	4	23	FOD
7	F	59	I, II, IV, and left hip joint	Hepatic infection	RD + HR + AHP + FPR + AC	2	21	FOD
8	F	14	I, II, IV, and right hip joint	None	RD + HR + AHP + FPR + AC	3	23	HP
9	F	30	I, II, III, IV, and left hip joint	Pulmonary infection	RD + HR + AHP + FPR + AC	2	13	FOD
10	M	64	I, II, and left hip joint	None	RD + AC	4	25	HP
11	M	42	I, IV, and left hip joint	None	RD + AC	6	24	FOD
12	M	32	I, II, IV, and left hip joint	None	RD + HR + AHP + FPR + AC	6	19	FOD
13	F	31	II and right hip joint	Pulmonary infection	RD + AC	5	22	FOD
14	M	51	I, II, and left hip joint	Hepatic infection	RD + FPR + AC	3	26	HP
15	M	26	I, II, and left hip joint	Pulmonary infection	RD + AC	3	19	FOD

AC = antiparasitic chemotherapy; AHP = artificial hemipelvic prosthesis; F = female; FOD = free of disease; FPR = femoral prosthesis replacement; HP = hip pain; HR = hemipelvis replantation; M = male; MSTS = Musculoskeletal Tumor Society; RD = radical debridement.

**Figure 1.**
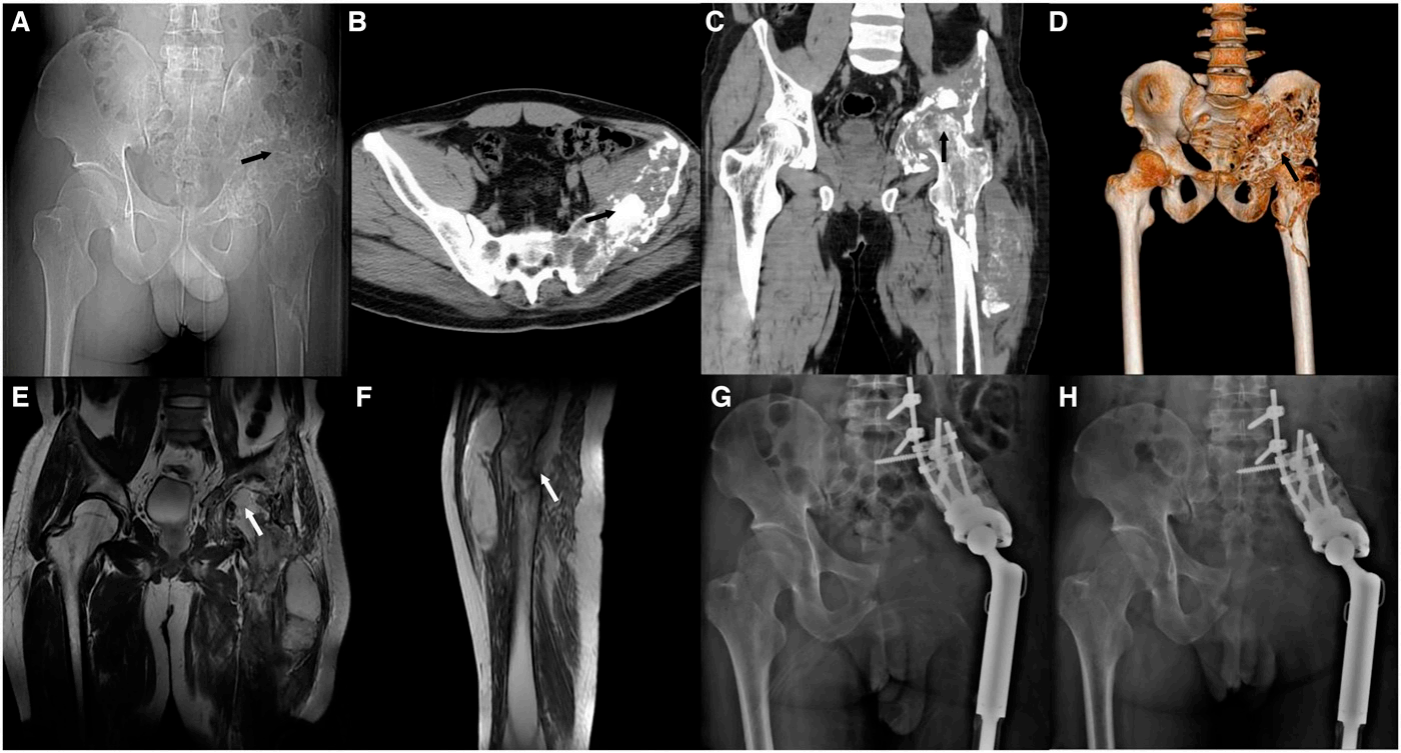
A 30-year-old man presented with progressive, painful limitation of the left hip for 6 months. (**A**) Preoperative anteroposterior X-ray of the pelvis showed osteolytic bone destruction in the left ilium, periacetabulum, pubic bone, ischium, and proximal femur, with interruption of cortical continuity in the left proximal femur (black arrow). (**B–D**) Preoperative computed tomographic scans and three-dimensional reconstruction showed multiple areas of low-density bone destruction in regions I, II, and III of the left pelvis and proximal left femur (black arrows). (**E, F**) Preoperative magnetic resonance images showed multiple, round, long T2 signals of different sizes in the left ilium with surrounding soft tissues, left pelvic wall, and left buttock muscle (white arrows). (**G, H**) Postoperative and postoperative month 3 anteroposterior X-rays of the pelvis showed good alignment of the femoral prosthesis and pelvic internal fixation.

**Figure 2.**
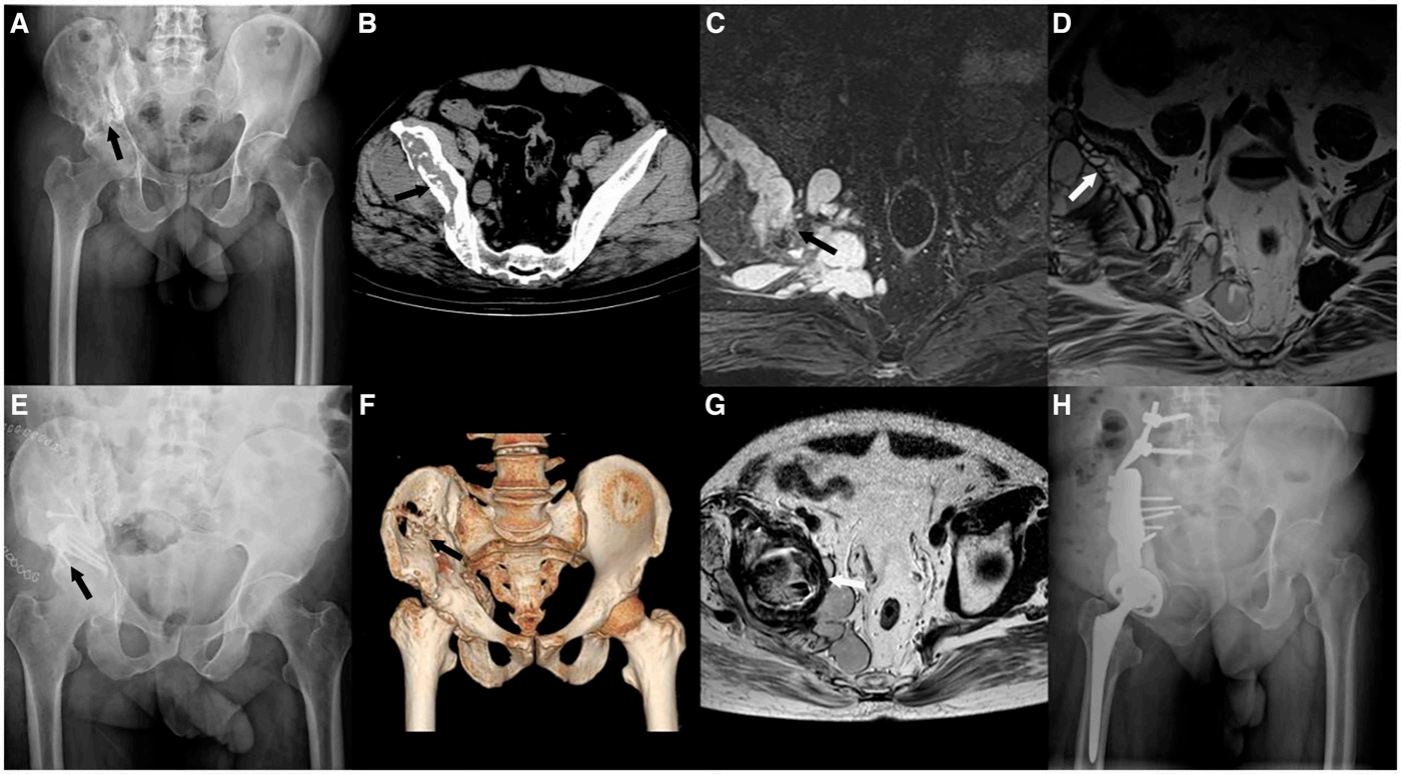
A 46-year-old man presented with right hip/groin pain, swelling, and a painful mass of the right hip. (**A**) Preoperative anteroposterior X-ray of the pelvis showed osteolytic bone destruction around the right ilium, sacroiliac joint, and acetabulum (black arrow). (**B–D**) Preoperative computed tomographic scan and magnetic resonance (MR) images showed bone destruction in regions I, II, and IV of the right pelvis and proximal right femur (black arrows and white arrow). (**E**) Anteroposterior X-ray of the pelvis after debridement with the bone defect filled by bone cement and fixation with a plate internal fixation (black arrow). (**F, G**) Computed tomographic three-dimensional reconstruction and MR image at postoperative year 2 after the first operation showed multiple areas of bone destruction around the right ilium, acetabulum, and upper femur, indicating signs of recurrence of infection (black arrow and white arrow). (**H**) Postoperative anteroposterior X-ray of the pelvis showed good alignment of the femoral prosthesis and pelvic prosthesis with stable pelvic reconstruction.

All patients received treatment with surgery combined with antiparasitic chemotherapy (Figures 1 and 2). The mean number of surgical interventions per patient for pelvic CE was 5.3 (range, 2–9 interventions per patient). At the final follow-up, the mean MSTS score of 15 patients was 21.73 ± 3.78 points, and the postoperative horizontal deviation distance of the acetabular rotation center of the prosthesis was 8.61 ± 4.36 mm, with a vertical deviation distance of 6.35 ± 2.92 mm. Eleven patients were able to walk spontaneously, whereas four required the use of a walking aid by the first postoperative month. Recurrence of pelvic echinococcosis was observed in five patients, two of whom had a hepatic infection. However, the infection did not develop further after repeated debridement procedures combined with more than 6 months of antiparasitic drugs. In addition, superficial wound skin necrosis of the surgical incision occurred in one patient, who recovered successfully after additional debridement.

## DISCUSSION

Pelvic hydatid bone disease has been reported to account for approximately one fourth of all cases of bone echinococcosis.^13^ Unlike visceral hydatid bone disease, the development of hydatid bone disease begins in blood-rich cancellous bone. In most cases, there is a lack of a connective tissue barrier against intraosseous hydatid cyst proliferation, allowing daughter cysts to enter the bone directly and grow in the bone marrow cavity along the direction of the epiphyseal plate and articular cartilage.^9^^,^^11^^,^^14^ On imaging, the lesion will appear expansively honeycombed or soap bubble–shaped, with smooth and sclerotic margins. This growth pattern of hydatid bone disease determines its cystic and multilocular features.^15^^,^^16^ The development of pelvic hydatid bone disease is slow and its symptoms are also often concealed, ranging from chronic pain, swelling, claudication, sinus formation, abdominal pain, or nerve compression symptoms (e.g., sciatica). Therefore, the diagnosis of pelvic hydatid bone disease should be comprehensive, taking into account factors such as living environment, serological tests (whole blood tests),^17^ parasite-specific antibody examinations (ELISA and DIGFA),^18^^,^^19^ imaging examinations, and clinical manifestations. This approach can help to avoid misdiagnosis or missed diagnosis, which may prevent the occurrence of delayed treatment and may worsen symptoms.

Currently, the primary method for treating advanced pelvic hydatid bone disease is surgical intervention combined with antiparasitic chemotherapy.^2^^,^^11^ Chemotherapy with mebendazole or albendazole alone tends to be insufficient in most patients; however, it can provide adjuvant therapy to reduce cyst range preoperatively, and adjuvant therapy reduces the risk of recurrence as well. Published studies^2^^,^^4^^,^^20^ have reported different surgical methods, including complete resection, total hip arthroplasty, femoral–pubic arthrodesis, and so on. In most cases, it is impossible to eradicate the advanced lesion without resulting in too much patient disability. The resection of large segmental bones usually leads to the loss of continuity and integrity of the pelvic ring. Thus, restoring pelvic continuity is of utmost importance after debridement for patients with lesion areas in more than half of the pelvic ring. Our study managed 15 patients using a designed hemipelvic prosthesis to restore the pelvic ring. All patients (100%) with compression symptoms caused by pelvic hydatid bone disease improved after surgery.

The treatment of hydatid bone disease is more similar to oncological therapy than the surgical treatment of visceral hydatid disease.^4^ However, pelvic hydatid bone disease cannot be fully equated with malignancy because it tends to be less aggressive. Therefore, the eradication of lesions using surgical treatment is not a complete treatment, but rather a primary step in the management of osseous echinococcosis. In most cases, the recurrence of infection or worsening symptoms may result from incomplete debridement surgery, indicating that preoperative and postoperative antiparasitic chemotherapy is an effective and essential step in the treatment protocol to control the spread and recurrence of cystic lesions.^9^^,^^11^ All patients in this study were treated successfully with surgery combined with antiparasitic chemotherapy. The mean MSTS score of 15 patients at the final follow-up was 21.73 ± 3.78 points. Unfortunately, five patients still experienced a recurrence of infection; however, the infection was controlled after repeated debridement procedures.

When osteolytic destruction of the pelvis is not extensive, some researchers suggest that hemipelvis devitalized replantation using liquid nitrogen is an optional method of reconstruction.^6^^,^^20^ However, it is difficult to select a well-integrated allogeneic hemipelvis for allogeneic hemipelvis replacement, and allogeneic reactions should not be overlooked. Both methods mentioned here may result in postoperative recurring fractures and reinfection. Campanacci et al.^21^ suggested using an acetabular prosthesis connected to a pedicle screw–rod system to restore the pelvic bone defect caused by bone tumor resection, which inserted screws on the sacral side and connected the pelvic acetabular prosthesis to the sacral screws through the pedicle screw–rod system. Zhang et al.^22^ reported a series of patients with pelvic bone defects treated with a new, modular artificial hemipelvic prosthesis, which was fixed to the sacrum with screws. In our study, the prosthesis combined with a sacral pedicle screw–rod system was designed anatomically according to preoperative CT scanning, providing a good fit with the sacral articular surface and effecting simple installation. Sacral screws can also be placed precisely in the sacrum based on the preoperative design, avoiding entering the sacral canal and the vascular plexus anterior to the sacrum, and thus reducing the risk of prosthesis dislocation, refracture, and nerve and vascular injury.

In summary, the treatment of pelvic hydatid bone disease is intricate, and surgeons should be aware that it is impossible in most cases to eradicate the disease without inducing too much disability. Palliative treatment with limb salvage surgery is an effective method for managing advanced pelvic hydatid bone disease. Several limitations may affect the results of our study. First, it is retrospective study conducted by a single medical institution with a small sample size. Second, there is no unified, standardized treatment algorithm for the management of pelvic echinococcosis. Therefore, we acknowledge that more patients and a longer follow-up are needed to evaluate the efficacy of this treatment method.
